# Ney’s Battery

**Published:** 1869-03

**Authors:** 


Ney's Battery.—This cheap and convenient battery is constructed as follows: It consists, in the first place, of a glass vessel, filled with a solution of sal-ammoniac; then of an amalgamated zinc plate, which dips into the vessel. Further, of a porous cylinder of clay, standing in the same vessel, and filled with carbonate of copper, in which a plate of copper is suspended. For field and telegraph purposes, it is well to fill the glass vessel with fine sand, saturated with sal-ammoniac solution, instead of the pure solution itself. It is only necessary to add the crystal sal-ammoniac from time to time, in order to keep this battery in constant activity. The carbonate of copper is itself insoluble in the sal ammoniac solution; but under the influence of the galvanic current, the sal-ammoniac is separated into the hydrochloric acid and ammonia, the first of which attaches itself to the zinc, and the latter to the salt of copper, and dissolves it. During the reduction, a secondary current is produced, which is equivalent in strength to that of a Daniell's battery.

